# The effects of dissection-room experiences and related coping strategies among Hungarian medical students

**DOI:** 10.1186/s12909-015-0355-9

**Published:** 2015-04-11

**Authors:** Imola Sándor, Emma Birkás, Zsuzsa Győrffy

**Affiliations:** Institute of Behavioural Sciences, Semmelweis University, Budapest, Nagyvárad tér 4, Hungary H-1089

**Keywords:** Dissection, Coping, Career socialisation, Hidden curriculum, Gender differences

## Abstract

**Background:**

Students get their first experiences of dissecting human cadavers in the practical classes of anatomy and pathology courses, core components of medical education. These experiences form an important part of the process of becoming a doctor, but bring with them a special set of problems.

**Methods:**

Quantitative, national survey (n = 733) among medical students, measured reactions to dissection experiences and used a new measuring instrument to determine the possible factors of coping.

**Results:**

Fifty per cent of students stated that the dissection experience *does not affect them*. Negative effects were significantly more frequently reported by women and students in clinical training (years 3,4,5,6). The predominant factor in the various coping strategies for dissection practicals is *cognitive coping* (rationalisation, intellectualisation). *Physical* and *emotional* coping strategies followed, with similar mean scores. Marked gender differences also showed up in the application of coping strategies: there was a clear dominance of emotional-based coping among women. Among female students, there was a characteristic decrease in the physical repulsion factor in reactions to dissection in the later stages of study.

**Conclusions:**

The experience of dissection had an emotional impact on about half of the students. In general, students considered these experiences to be an important part of becoming a doctor. Our study found that students chiefly employed cognitive coping strategies to deal with their experiences.

Dissection-room sessions are important for learning emotional as well as technical skills. Successful coping is achieved not by repressing emotions but by accepting and understanding the negative emotions caused by the experience and developing effective strategies to deal with them.

Medical training could make better use of the learning potential of these experiences.

## Background

Anatomy and pathology are central subjects in medical training, and the practical classes for these provide students with their first dissection-room experiences.

Several studies have addressed the effect on medical students of dissection-room experiences [1-8]. These experiences are found to play a part in learning how to handle emotions as well the subject itself, and the effects can carry over to the subsequent doctor-patient relationship [6,9]. There have been many studies worldwide on the role of dissection in medical career socialization and professionalization [2,10]. In one much-cited study, Hafferty calls the experience of dissection an emotional rite of passage which promotes the process of changing over from layperson to doctor [2]. This ritual takes place via a psychological process of which very little is yet known [11]. Students’ encounters with the subject of death and dying and the revelation of the inside of the body on these practicals is a “licence to intrude”, a situation which – especially considering their age – they may not be prepared for, while successfully coping with it can be a key element of their subsequent medical careers.

Studies of dissection-room experiences consider several phenomena. Those concerned with the stress-arousing nature of the experiences report contradictory results. Some find that students quickly adapt to the experiences [4,12,13] and tend to regard them as a challenge [14]. Other studies find that students tend to react negatively to the experience [15]: stress heightened by intensive psychological and physical reactions, sleep disorders [16], and intensive anxiety [17], which can reach the level of post-traumatic stress [18].

Much has been written on the stress-arousing nature of dissection-room experiences and on coping with them. One study describes medical students’ successful coping with dissection-room experiences as a “Rubicon”, a measure of fitness for a medical career [3]. This is a belief which develops only latently, as part of the hidden curriculum [19]. Accordingly, anybody suffering from these experiences is likely to keep the fact quiet, fearing stigmatization and being “declared unfit”. Behind this lies the stereotypical view that anybody unable to bear these testing experiences is not suited to the doctor’s calling. Consequently, coping at dissection practicals is considered successful if the student shows no feelings at all. To behave otherwise is incompatible with strictly scientific, objective professional behaviour [20]. The teaching of the subject does not cover, or hardly covers, what might be regarded as normal and abnormal reactions, how the experiences affect different people, or how the uncomfortable physical and psychic reactions to the situation might be coped with [21]. Students try to “pick this up” from each other and from their role-model teachers and health-care staff. In many cases, however, they find other people’s conduct unacceptable (e.g. humour, lack of respect for the dead) and neither can they accept their own emotional reactions. In general, emotional reactions to the situation are regarded as a personal, private matter, and students largely have to cope with it on their own [22].

Silver compared the psychological process of medics’ career socialisation with the battered child syndrome, citing episodes of abuse and neglect to which students are subjected, gradually turning their initial enthusiasm into depression or fear, as with abused children [23]. Another writer has compared medical education to a neglectful, abusive family characterised by high demands, denial, indirect communication patterns, isolation and rigidity [24].

Opinions are also divided as to whether there are significant gender differences in the effects of dissection-room experiences. Some studies have found no gender differences in the impact of dissection [25]. Several others, however, have reported higher levels of stress among women than men [7,25]. Of course this may have been due to women being more prepared to admit and divulge their own emotional reactions [25]. Gender differences have been found most frequently in the degree of anxiety and the mode of coping [7,26,27].

Another focus of studies is the effect of the students’ background and past experience on their mode of coping. This has produced the finding that previous knowledge and experience of death and dying help students to cope, while negative events currently in progress (grief, loss) tend to reinforce the effect of the experiences and hinder coping [28].

In Hungary, students’ dissection-room experiences come from their studies of anatomy in the first two years, pathology in the third year and forensic medicine in the fifth year. Students attend dissection room practicals totalling 152 hours in the four-semester anatomy course, 56 hours in the two-semester pathology course, and eight hours in the one-semester forensic medicine course. Practicals usually take place in groups of 15–20 students, and the gender ratio is 60 females to 40 males. The students do not usually do the dissection themselves, although they may volunteer to do so. Preparation before practical sessions is usually unstructured, and only rarely touches on how to manage emotions. There is no organised discussion or reflection on dissection experiences. It is important, however, that at the end of the year, the students may voluntarily take part in a ceremony to pay respects to the dead.

The purpose of the present study was to survey the impact of the dissection-room experiences among medical students in the Hungarian education system, the modes of coping employed by the students, and any gender differences involved. We wanted to discover whether there are any changes among the various episodes of medical training in this respect, and to discern Hungarian medical students’ attitudes to their dissection experiences. No such research covering students at different stages of their course and jointly examining the effects of several subjects (anatomy, pathology, forensic medicine) had previously been conducted. In this respect, our study was unique.

## Methods

In academic year 2012/2013, we carried out an anonymous, self-completion paper based questionnaire survey of students on the general medicine course in the four medical universities in Hungary (Budapest, Debrecen, Pécs and Szeged). The main selection criterion was that the sample should include equal proportions of students in preclinical and clinical training. To judge from the literature [29], the “watershed” periods are the 1st, 3rd and 6th years, and we attempted to include as many students from these as possible. For data protection reasons, we were unable to use probability sampling for the questionnaire survey. The survey was carried out under permission granted by the Semmelweis University Ethics Committee (TUKEB permit 59/2013).

### Ethical approval

The study was approved by the Ethics Committee of the Semmelweis University, Budapest.

The research centred on assessing the physical and mental health, health behaviour, stress load, coping strategies and career motivation of Hungarian medical students. The design of the questionnaire for the study was largely based on previous research on doctors [8,29,30].

### Measuring instruments

The survey questions were grouped in six major topics (Table 1).

**Table 1:** **The survey questions were grouped as follows**

1.	Demographic data (sex, age, year, university).
2.	Health data (psychosomatic symptom list, health self-assessment).
3.	Psychological factors (depression, sleep disorders, suicidal behaviour, burnout, empathic attitude, parental attachment).
4.	Health behaviour (smoking, alcohol and tranquiliser consumption, sport).
5.	Vocational background factors (doctor parents, time of career choice, career-choice motivations).
6.	Sources of stress during university years (stress factors, examination stress, overload, coping, dissection-related sources of stress).

Dissection-related experiences and modes of coping were measured using the following questions (students chose from a list of possible answers those most applicable to themselves):

1. What sources of stress are there in your life?

Possible answers: “study workload”, “concentration on study”, “university staff”, “examinations”, “pressure of time”, “practicals involving dissection”, “parent-/partner-/friend-related stress”, “own or relative’s illness”, “financial- or future-related sources of stress”.

2. What are your typical reactions to the dissection experience? (multiple choice answers)

1. I often dreamt about it
2. I often thought about it afterwards
3. I had daytime flashbacks of it
4. I was afraid of being alone
5. I constantly talked about it
6. I turned inwards and did not speak to anybody about the experience
7. I started to doubt my fitness for a medical career
8. I can’t remember
9. It had no effect on me

3. The dissection experience was assessed by 17 statements, scored between a scale from 1 (I do not agree at all) to 5 (I fully agree) (Turcsányi) [31] (Table 2).

**Table 2:** **Percentage distribution of responses to dissection experiences in the full sample**

Scale from 1 (I do not agree at all) to 5 (I fully agree)	1	2	3	4	5
	Percentage of respondents				
1. I treat the cadaver as an object when dissecting	10.8	11.6	26.6	27.7	23.4
2. When dissecting, I try to make myself believe that the cadaver is not a person, but only resembles one.	54.3	16.2	12.0	10.2	7.3
3. I look at the cadaver as if it was plastic, or a dummy.	55.4	15.2	11.6	10.3	7.4
4. When dissecting, I cannot look on the cadaver as if it was a dummy.	17.3	12.1	18.7	25.5	26.4
5. When dissecting, I do not think that the corpse is a person, just flesh and bone, like in the kitchen.	35.5	16.1	19.7	15.4	13.4
6. In difficult situations it is important to maintain objectivity and the right distance.	2.7	3.5	17.4	30.1	46.3
7. In operations and dissection, I pay attention to the illness and pathological phenomena, and surgical and dissection technique, rather than the person.	3.3	6.2	18.3	35.0	37.3
8. To attain my aims, I had to go through such experiences and learn all of this.	2.0	2.3	8.0	19.4	68.4
9. I concentrate on problem-solving, not the visual impression.	2.1	4.2	12.5	30.7	50.4
10. You have to accept that pain, suffering and death are all part of life.	2.1	1.8	6.2	23.8	66.1
11. If such an emotional thought creeps in, I put a stop to it.	31.3	20.4	19.4	16.7	12.2
12. I try to avoid looking into the cadaver’s face and eyes.	38.0	18.1	16.0	14.3	13.7
13. I take the view that others have put up with it, and so will I.	42.2	12.0	14.6	14.9	16.3
14. A good mood and humour are very important at dissection-room practicals.	15.0	19.3	29.8	23.9	12.0
15. Humour and levity are not appropriate for dissection.	30.2	24.4	20.9	15.3	9.1
16. Touching a cadaver is never a problem.	8.0	9.1	14.9	23.7	44.3
17. Neither do I have any difficulty if I see a baby, child or young person on the dissection table.	36.6	22.3	19.5	11.8	9.8

1. I treat the cadaver as an object when dissecting.
2. When dissecting, I try to make myself believe that the cadaver is not a person, but only resembles one.
3. I look at the cadaver as if it was plastic, or a dummy.
4. When dissecting, I cannot look on the cadaver as if it was a dummy.
5. When dissecting, I do not think that the corpse is a person, just flesh and bone, like in the kitchen.
6. In difficult situations it is important to maintain objectivity and the right distance.
7. In operations and dissection, I pay attention to the illness and pathological phenomena, and surgical and dissection technique, rather than the person.
8. To attain my aims, I had to go through such experiences and learn all of this.
9. I concentrate on problem-solving, not the visual impression.
10. You have to accept that pain, suffering and death are all part of life.
11. If such an emotional thought creeps in, I put a stop to it.
12. I try to avoid looking into the cadaver’s face and eyes.
13. I take the view that others have put up with it, and so will I.
14. A good mood and humour are very important at dissection-room practicals.
15. Humour and levity are not appropriate for dissection.
16. Touching a cadaver is never a problem.
17. Neither do I have any difficulty if I see a baby, child or young person on the dissection table.

We compiled the questions on the basis of two preliminary studies [31]. In the first, 25 students wrote in their own words what they do to get through dissection sessions. This resulted in 22 questions, which we further reduced via a factor analysis on a sample of 246 medical students.

In the present study, we used the 17 questions which proved relevant in the preliminary studies and the three subscales derived from them, and then tested the internal consistency. The subscale determining treatment of the body as an object (objectification subscale) contains the first 5 questions, where the 4th question is reversed; Cronbach alpha: 0.71. (E.g. I look at the cadaver as if it was plastic, or a dummy).

The questions examining general cognitive attitude (cognitive subscale) are numbers 6 to 10, and also showed acceptable internal consistency; Cronbach alpha: 0.67. (E.g. 8. To attain my aims, I had to go through such experiences and learn all of this).

The third subscale measures coping with emotions (emotions subscale), involving questions 11, 12 and 13, and the reversed questions 16 and 17. Its Cronbach alpha is 0.72. (E.g. 12. I try to avoid looking into the cadaver’s face and eyes).

Questions concerning humour severely spoiled the internal consistency of the emotional subscale, and so were not used in subsequent analyses.

The socio-demographic variables used in the analysis were: the two gender categories and the categories of preclinical (1st and 2nd year) and clinical (3rd to 6th year) students. Preclinical students obtain their dissection experiences in anatomy practicals, and clinical students in pathology - and possibly forensic medicine – practicals.

### Statistical methods

The descriptive analyses determined frequency, mean and variance. We also looked at percentage differences between the measured variables. Depending on the variable type, we used ANOVA, independent-sample *t*-test and chi-squared test. This paper analyses the responses of medical students (n = 733) and in every case we checked the proportions of valid answers. Statistical analysis of the data was performed using the SPSS 15.0 program.

## Results

The study involved questioning 18.78% of all Hungarian medical students (733). The gender distribution in the sample was 33.2% men (243) and 66.8% women (488), which corresponds to the average gender ratio in Hungarian medical training according to Central Statistical Office (KSH) data [32]. About half each of the sample were studying in the preclinical (1st and 2nd years, 48%) and clinical periods (3rd and 6th years, 52%) (Table 3). The mean age of students in the sample was 22.4 years (SD = 2.14). The distribution by medical university was 47.1% (345) Budapest, 4.5% (33) Debrecen, 26.2% (192) Pécs and 22.2% (163) Szeged.

**Table 3:** **Distribution of students by year of study, age and gender**

	Men		Women		Full	
	N	Age Mean (SD)	N	Age Mean (SD)	Age N Mean (SD)	
Preclinical group	119	21.34 (1.83)	227	21.06 (1.38)	346	21.15 (1.55)
Clinical group	120	23.72 (1.88)	255	23.73 (1.84)	375	23.72 (1.85)

**Figure 1:**
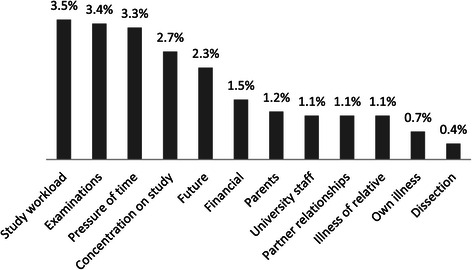
Main sources of stress given by students.

The first source of stress to be appraised was the effect of dissection. For the responding students, the most important sources of stress were the study workload, the burden of examinations and the pressure of time. These were followed by difficulty of concentrating on study, worries about the future, and financial anxieties. Then came worries about private life (partner relationships, relatives, illness). Figure 1 Although very few students identified participation in dissection practicals as a source of stress, there were distinctive differences between men and women in the assessment of dissection practicals: a significantly larger proportion of women felt these subjects to be “stressful” (1.2% vs. 6.4%; *χ*^2^(1) = 9.79 P = 0.00). There were differences on the effect of dissection experience between genders and between different years. A significantly larger proportion of women in both year-groups stated that the dissection experience had an effect on them (preclinical group: *χ*^2^(1) = 14.81; P = 0.00; clinical group: *χ*^2^(1) = 20.04; P = 0.00). Among women, this was more frequently true in the clinical group (*χ*^2^(1) = 7.71; P = 0.00), while no significant difference by year of study was found among men (Table 3).

**Figure 2:**
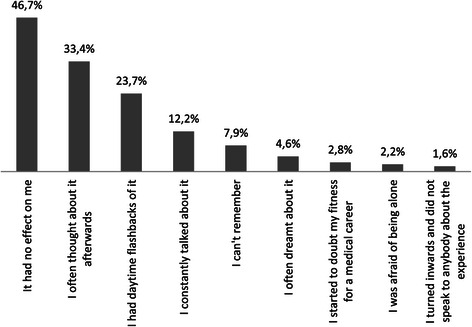
Frequency of reactions to dissection practicals.

In the next step, we examined the possible reactions to dissection practicals. About 50 per cent of students stated that the dissection experience “had no effect” on them, while for 33.4 per cent, it “I often thought about it afterwards” and 23.7 per cent said, “I had daytime flashbacks of it” (Figure 2).

Agreement with the statements “I often thought about it afterwards” and “I had daytime flashbacks of it” was significantly more frequent among women in the clinical group than women in the preclinical group (*χ*^2^(1) = 8.35; P = 0.00 and *χ*^2^(1) = 5.87; P = 0.02), and in the clinical group, women were more likely than men to report these experiences (*χ*^2^(1) = 15.76; P = 0.00 and *χ*^2^(1) = 10.36; P = 0.00). Table 4 shows that women reported almost twice as frequently as men that “I often thought about it afterwards” and “I had daytime flashbacks of it”.

**Table 4:** **Yes-responses to dissection experiences broken down by gender and year of study**

	Male N (%)		Female N (%)		*χ*^ 2 ^ (1)		Preclinical N (%)		Clinical N (%)		*χ*^ 2 ^ (1)
*It had no effect on me*											
Clinical	79	(66.9)	101	(45.1)	14.81**	male	79	(66.9)	67	(57.3)	ns.
Pre-clinical	67	(57.3)	82	(32.7)	20.04**	female	101	(45.1)	82	(32.7)	7.71*
*I often thought about it afterwards*											
Clinical	28	(23.7)	71	(31.7)	ns.	male	28	(23.7)	27	(23.1)	ns.
Pre-clinical	27	(23.1)	112	(44.6)	15.76**	female	71	(31.7)	112	(44.6)	8.35*
*I had daytime flashbacks of it*											
Clinical	17	(14.4)	50	(22.3)	ns.	male	17	(14.4)	19	(16.2)	ns.
Pre-clinical	19	(16.2)	81	(32.3)	10.36*	female	50	(22.3)	81	(32.3)	5.87*

*p < 0.05, **p < 0.001.

In the final step, we examined the different modes and strategies of coping with dissection practicals. The highest scoring of the three subscales was found to be the cognitive subscale, followed with roughly similar scores by the objectification and emotional subscales (Table 5).

**Table 5:** **Means and variances of dissection-experience coping subscales**

	Mean	SD
Objectification subscale	12.6	4.6
Emotional subscale	13.4	4.8
Cognitive subscale	21.4	3.1

**Figure 3:**
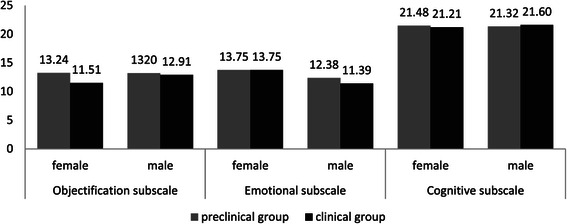
Means of objectification, emotional and cognitive subscales by year of study and gender.

The effects of gender and year of study on the objectification, cognitive, and emotional subscales were tested by independent ANOVAs. Gender and year-groupings exerted a significant interactional effect on the objectification subscale (F(1) = 3.86; P = 0.05; η^2^ = 0.006). Post hoc analysis by *t*-test showed significant differences between year-groups among women (t(454) = 4.03; P < 0.00). The mean in the clinical group was significantly higher among men (t(352) = 2.80; P < 0.00). The women in years 3 and 4 were thus least likely to view the cadaver as an object (Figure 3).

Results also shows that women gave significantly higher scores on the emotional coping subscale than men (F (1) = 36.6; P = 0.00; η^2^ = 0.05) (Figure 3).

We did not examine the effect of university towns on the dissection questionnaire subscales. This was because the medical syllabus is traditionally common among all university towns, so that no attempt was made to include similar proportions of preclinical and clinical students in the samples in different universities. Consequently, the distribution of students by year of study was not homogeneous among the towns (Khi^2^ (3) = 112,650 p = 0.00) and the effect of university towns could not be examined reliably.

The cognitive coping subscale showed no differences by either gender or year of study (Figure 3).

## Discussion

Dissection-related experiences and coping strategies of Hungarian medical students were investigated in a national survey of students in academic year 2012/2013. Among the sources of stress identified by medical students, dissection practicals were the least mentioned (Figure 1).

Nearly 50 per cent of the students who took part in our study stated that dissection practicals “had no effect” on them. There were distinctive differences in responses among students of different gender and in different years of study: men reported to a significantly greater degree that the dissection experience had no effect on them, while women were twice as likely to state, “I often thought about it afterwards” and “I had daytime flashbacks of it”. In line with these results, we found that women reported to a significantly greater degree that dissection practicals were a source of stress for them. There was also a significant difference between the experiences of students in preclinical and clinical training. First- and second-year students were significantly more likely to state that the experience of dissection “had no effect” on them, and less likely to state that they often thought about, or had flashbacks of, the experience of dissection practicals.

In terms of the psychometric indicators, our measuring instrument for strategies of coping with dissection practicals, designed in multiple steps, proved acceptable. The dominant factor in the sample was “cognitive coping” (rationalisation, intellectualisation), followed – with similar mean scores – by objectification and emotional coping. The distinctive differences by gender also showed up strongly in the choice of coping strategies: among women, there was a clear dominance of emotion-based coping. In the later years of study, however, female students displayed a definite decrease in the physical repulsion factor in reactions to dissection.

Our study found that students regarded dissection-room experiences as essential. Nearly 87% agreed with the statement, “to attain my aims, I had to go through such experiences and learn all of this”. Table 2 This corresponds with findings in the international literature that students see dissection-room experiences as important for their career socialisation, primarily because they thereby learn appropriate ways of handling their own emotional reactions and thus become better able to cope with the kind of difficult, emotionally-burdening situations they will have to deal with later [3].

The work is novel in identifying units of analysis in medical students’ experiences of dissection of the human body at different stages of their training. Our measuring instrument was designed to distinguish modes of coping and thus throw light on their heterogeneity. The differences in the impact of dissection-room experiences on students in preclinical and clinical years could be explained by the process character of the phenomenon. Successful adaptation involved a gradual shift from the initial, over-reserved attitude to a more mature, “healing” attitude. Further study will be required, however, to understand and explain this phenomenon fully.

The fact that women react more sensitively than men to dissection-room experiences may have long-term consequences for one of the central issues in medicine today, the feminisation of the profession. In the 1990s, the proportion of women among doctors was forecast to rise to one in three by 2010; in reality, women now account for more than half of those choosing and following a medical career [33,34]. The trend is strongly apparent in Hungary. Figures from the Central Statistical Office show that there are twice as many women than men in the young-doctor age group (26–29 years) [32]. Helping female students to cope with their higher sensitivity to stress in dissection practicals should be made part of medical training.

Our results demonstrate the presence of coping and the predominance of cognitive coping across gender and stage in training. The problem is to identify real “success”. An approach based on reservation and repressed emotions may seem appropriate in the situation if it helps the student to “get through” practical sessions. What is not clear, however, is whether this can develop into a general pattern the future doctor’s medical practice. An over-reserved attitude to patients and impersonal, objective behaviour are frequently cited as signs of burnout [35]. There are important questions concerning the extent to which this behaviour arises from socialisation during students’ university careers, how large a part is played by the emotions evoked by their dissection experiences and the ways they find to handle these experiences, and whether ways can be found to help adaptive coping strategies. For the purpose of this study, we regard successful coping as the ability to handle emotions and behaviours connected to the situation, whatever the initial difficulties (crying, evasion, etc.). Furthermore, the dissection room experiences should have a positive effect on their clinical attitudes, i.e. they should become capable of integrating the negative emotions caused by the experience by becoming aware of and understanding them, and developing effective strategies to deal with them.

## Conclusions

The methodology of anatomy teaching has frequently been a focus of interest in recent decades. The need for dissection practicals in the usual form has been called into question in the light of technical developments [36]. Those in favour of reforming dissection practicals argue that substituting for real cadavers could alleviate students’ negative reactions [3]. In one British university, students learn anatomy using imaging techniques and dummies instead of dissecting real cadavers [37]. This practice has not become widespread, however, because most researchers and educators agree that practical dissection is invaluable as a career-socialisation experience, and recommend that the general curriculum should retain dissection in its customary form [9,38,39]. The difficulties faced by students in going through dissection-room experiences, however, is a problem which has to be addressed. Students are often unprepared for their own or their peers’ intense emotion reactions, and for the behaviour of their teachers and other health staff. For example, the emotionless approach of an anatomy lecturer may induce an over-reserved attitude which may carry over to the later doctor-patient relationship [12].

Investigations into these phenomena have given rise to some recommendations in the international literature for possible ways of alleviating the harmful effects and helping students to cope. Weeks, Harris and Kinzey in their study [40] make four recommendations for helping students in their study of anatomy. First, they urge that the language used in the dissection room should be respectful and be a “carrier” of a more humane attitude. Accordingly, they recommend the use of the word “donor” instead of “cadaver”. They also consider it important to reveal personal information about the donor (name, age, history, cause of death), which can help students in relating to the dead body as a previously-living human being. Thirdly, they recommend that students should talk about the thoughts and feelings aroused by the dissection and reflect on their experiences. Fourthly, they propose a commemoration ceremony as a positive ending to an emotionally and intellectually demanding course. Another study has shown that involving more senior (third-year) students in teaching reduces negative psychological and emotional reactions to dissection [4,41].

Our work has opened up several possible lines of research. In future studies, we would like to investigate the possible role of these dissection-room experiences in the causation of burnout, sleep disorders and somatic symptoms, and to find ways of optimizing the effects of dissection-room experiences. Our research results suggest that dissection experiences could have an important role in the process of becoming a doctor. Students’ experiences and coping can indirectly influence the formation of the doctor-patient relationship and thereby have an effect on morbidity in the general population [42,43].

One limitation of our study is that we did not have detailed statistical socio-demographic data on the students to weight the results for representativeness, and this restricts the generalisation of the results. A strength of the study, however, is the production of a measuring instrument for coping with dissection-room experiences. Another strength is the involvement of students at different stages of study, so that the data was basically analysed from a social-science perspective. Both the development of the measuring instrument and the analysis over several course years hold out the potential for understanding the complexity of the process.
